# Anti–coronavirus disease 2019 (COVID‐19) targets and mechanisms of puerarin

**DOI:** 10.1111/jcmm.16117

**Published:** 2020-11-26

**Authors:** Xingyue Qin, Chen Huang, Ka Wu, Yu Li, Xiao Liang, Min Su, Rong Li

**Affiliations:** ^1^ Department of Neurology (Area Two) Guigang City People's Hospital The Eighth Affiliated Hospital of Guangxi Medical University Guigang China; ^2^ The Center for Data Science in Health and Medicine Business School Qingdao University Qingdao China; ^3^ Department of Pharmacy The Second People's Hospital of Nanning City The Third Affiliated Hospital of Guangxi Medical University Nanning China; ^4^ Guangxi Key Laboratory of Tumor Immunology and Microenvironmental Regulation Guilin Medical University Guilin China

**Keywords:** COVID‐19, molecular docking, network pharmacology, puerarin, targets

## Abstract

The present study aimed to uncover the pharmacological function and underlying mechanism of puerarin as a potential treatment for COVID‐19, using an in silico methodology, including network pharmacology and molecular docking. The pivotal targets of puerarin to treat COVID‐19 were identified and included the epidermal growth factor receptor (EGFR), tumour necrosis factor (TNF), tumour protein p53 (TP53), caspase 3 (CASP3), RELA proto‐oncogene (RELA), Fos proto‐oncogene (FOS), caspase 8 (CASP8), prostaglandin‐endoperoxide synthase 2 (PTGS2), interleukin 2 (IL2), protein kinase CB (PRKCB), B cell lymphoma/leukaemia gene‐2 (BCL2), protein kinase CA (PRKCA), nitric oxide synthase 3 (NOS3) and peroxisome proliferator–activated receptor gamma (PPARG). Functionally, the anti–COVID‐19 action of puerarin was associated with the suppression of oxidative stress and inflammatory cascades, and cell apoptosis. The signalling pathways of puerarin to treat COVID‐19 included modulation of the pathways of apoptosis, IL‐17 signalling, mitogen‐activated protein kinase (MAPK) signalling and TNF signalling. Molecular docking data illustrated the binding capacity of puerarin with COVID‐19 and the effective anti–COVID‐19 activity of puerarin. Taken together, our current network pharmacology–based findings revealed the pharmacological role of puerarin in the treatment of COVID‐19. Furthermore, the bioinformatic findings elucidated that some of these pivotal targets might serve as potential molecular markers for detecting COVID‐19.

## 1 INTRODUCTION

The severe acute respiratory syndrome coronavirus 2 (SARS‐CoV‐2) is the pathogenic agent responsible for causing COVID‐19, a severe and life‐threatening epidemic disease that has led to a massive economic burden and increasing death toll in the world.^1^ As an evolving epidemic, COVID‐19 is rapidly growing and spreading in several countries, including the United States, Brazil and India.^2^ Epidemiological evidence suggests that the trend of COVID‐19 infection is difficult to control because of insufficient management against COVID‐19, characterized by huge rates of cases in different countries.^3^ In the United States, COVID‐19 has already affected millions of people and is characterized by an increasing incidence and death rate.^4^ Pathologically, the main clinical characteristics of COVID‐19 are related to inflammation, cytokine storms, immunodeficiency and infection.^5^ In clinical management against COVID‐19, the common therapy may involve supportive care,^6^ such as breathing assistance, as the development of an effective drug and new vaccine is still ongoing.^7^ Traditionally, *Pueraria montana* (*Gegen*) is found to relieve muscle fever, neutralize alcoholism‐related hypoglycaemic and hypolipemic actions.^8^ Puerarin, rich in *Pueraria montana*, exerts evident pharmacological benefits, such as preventing coronary heart disease and anti‐alcoholism and anti‐diabetic effects.^9^ In our previous preclinical studies, puerarin was found to have potent hepatoprotection, neuroprotection and anti‐dysmetabolism effects.^10^, ^11^, ^12^, ^13^ It has been reported that puerarin may function as a natural amoebicidal agent to manage *Acanthamoeba*‐induced pneumonia.^14^ In addition, puerarin plays an important role in anti‐inflammatory action, including prevention of acute lung injury by reducing inflammatory stress.^15^, ^16^ However, there is currently no specific drug for the treatment of COVID‐19. Although some antiviral drugs and traditional Chinese medicines have been used in the clinical treatment of COVID‐19, its therapeutic effectiveness is not clear. Collectively, we aimed to determine and identify the curative effect of puerarin in the treatment of COVID‐19 and the therapeutic mechanisms involved. It is of great interest to use bioinformatic and computational methods based on network pharmacology to unveil the potential agent to treat illness.^17^ Using the network pharmacology strategy, we have reported the pharmacological targets and molecular pathways of bioactive agents against complex diseases.^18^, ^19^ Thus, in this bioinformatic report, we intended to reveal the component‐target‐pathway network and pharmacological mechanism of puerarin in the treatment of COVID‐19.

## 2 MATERIALS AND METHODS

### 2.1 Screening of the target of puerarin in the treatment of COVID‐19

The total established target genes of puerarin were selected using the effective tools of traditional Chinese medicine systems pharmacology (TCMSP), Swiss Target Prediction and SuperPred. Meanwhile, other COVID‐19–nosopoietic genes were acquired using the gene‐disease associations (DisGeNET) and GeneCards databases. Additionally, these acknowledged genes of puerarin and COVID‐19 were mapped prior to emendation using the UniProt tool. After functional enrichment analysis using FunRich software, all anti‐COVID‐19 biotargets of puerarin were screened and identified, as reported elsewhere.^20^, ^21^

### 2.2 Screening of pivotal targets of puerarin to treat COVID‐19 and construction of PPI network

The total acknowledged targets of puerarin and COVID‐19 were used to further determine and construct a functional protein association network via the STRING database following a specific algorithm. In addition, these merged targets of puerarin and COVID‐19 were used to construct a network of protein‐protein interactions (PPI) using the Cytoscape software. As revealed by topological parameters using the Network Analyzer tool, the pivotal targets of puerarin to treat COVID‐19 were identified and visualized.^22^, ^23^

### 2.3 Enrichment analyses in biological processes and Kyoto Encyclopedia of Genes and Genomes (KEGG) pathway of intersection targets

Using the R language packages, such as ‘ClusterProfiler’, ‘ReactomePA’, ‘org.Hs.eg.Db’ and ‘GOplot’ in the R language (3.6.1), the enrichment analysis and visualization of the biological process and KEGG pathways of the intersection targets were performed and obtained accordingly. Moreover, the information on gene annotation resulted from ‘org.Hs.eg.Db’, p‐value cut‐off = 0.05, *q*‐value cut‐off = 0.05 for enrichment prior to plotting the corresponding bubble chart, histogram and Circos circle chart.^24^, ^25^

### 2.4 Molecular docking analysis

Using the chemical‐protein binding method, the pivotal targets were screened out and identified for puerarin‐based molecular docking analysis. After searching for specific proteins through the PDB database, 5R84 protein was selected to dock with the puerarin compound. The ChemBio3D Draw in Chem Bio Office 2010 software was used to conduct the three‐dimensional structure of puerarin before docking the molecular structures using AutoDock Vina software. The rationality of the docking parameter setting was assessed according to the root‐mean‐square deviation (RMSD) of the ligand molecule. The RMSD ≤ 4 Å was the threshold for the conformation of the ligand molecule.^26^, ^27^

## 3 RESULTS

### 3.1 Findings of putative genes of puerarin and COVID‐19

**FIGURE 1:**
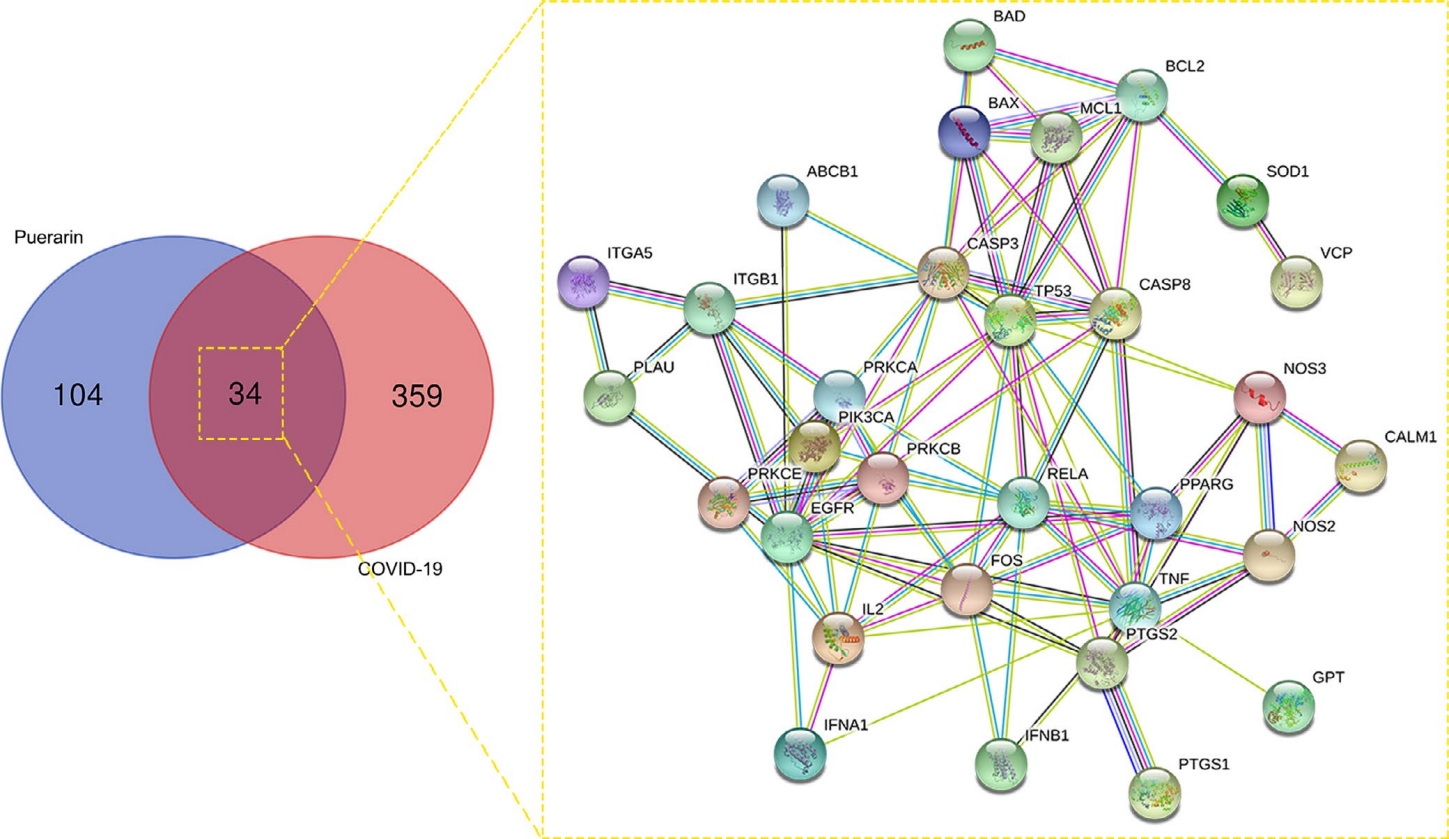
All common genes of puerarin and COVID‐19 were identified in a Venn map prior to further identification of the shared targets of puerarin to treat COVID‐19. Total 34 shared targets of puerarin to treat COVID‐19 were harvested and plotted for construction of a PPI network

As revealed in Figure 1, 393 COVID‐19–morbid genes were selected and detected. A total of 138 putative genes of puerarin were specified following the databases. As shown in the Venn diagram, 34 shared genes of puerarin to treat COVID‐19 were screened and identified. Furthermore, these mapped genes were reused to plot an interaction network.

### 3.2 Identification of all pivotal targets

**FIGURE 2:**
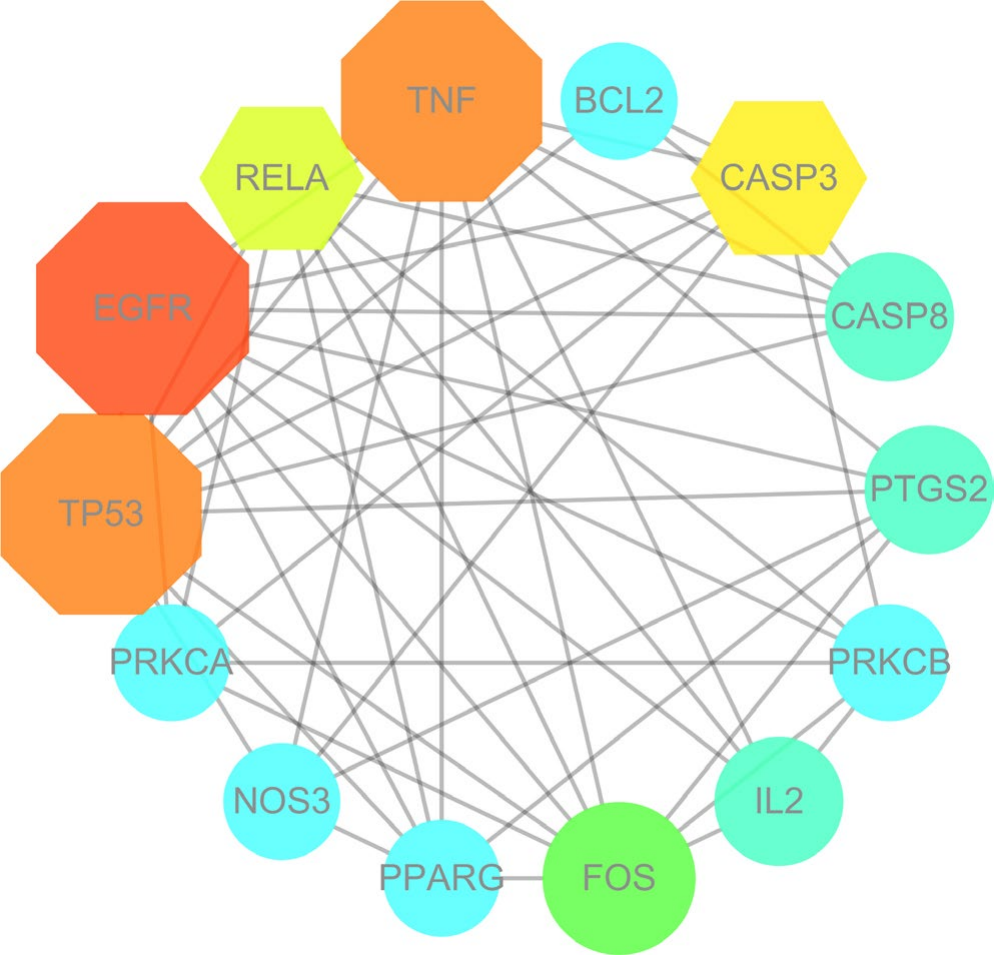
In further investigation, a total of 14 pivotal targets of puerarin to treat COVID‐19 were screened and identified. Meanwhile, these key genes were connected collectively in a functional map

All the mapped intersection proteins were input into Cytoscape 3.7.1 software to calculate the topological parameters of the interaction network of targets and functionally related proteins of puerarin in the treatment of COVID‐19. The algorithm from the network freedom median degree of 6.129 and freedom maximum degree of 15 was used to identify the pivotal targets of puerarin to treat COVID‐19. Therefore, the pivotal target screening condition range was set to 7‐15, and finally, 14 pivotal targets were obtained. These genes included *EGFR*, *TNF*, *TP53*, *CASP3*, *RELA*, *FOS*, *CASP8*, *PTGS2*, *IL2*, *PRKCB*, *BCL2*, *PRKCA*, *NOS3* and *PPARG*, as shown in Figure 2 and Table S1.

### 3.3 Enrichment findings of pivotal targets

**FIGURE 3:**
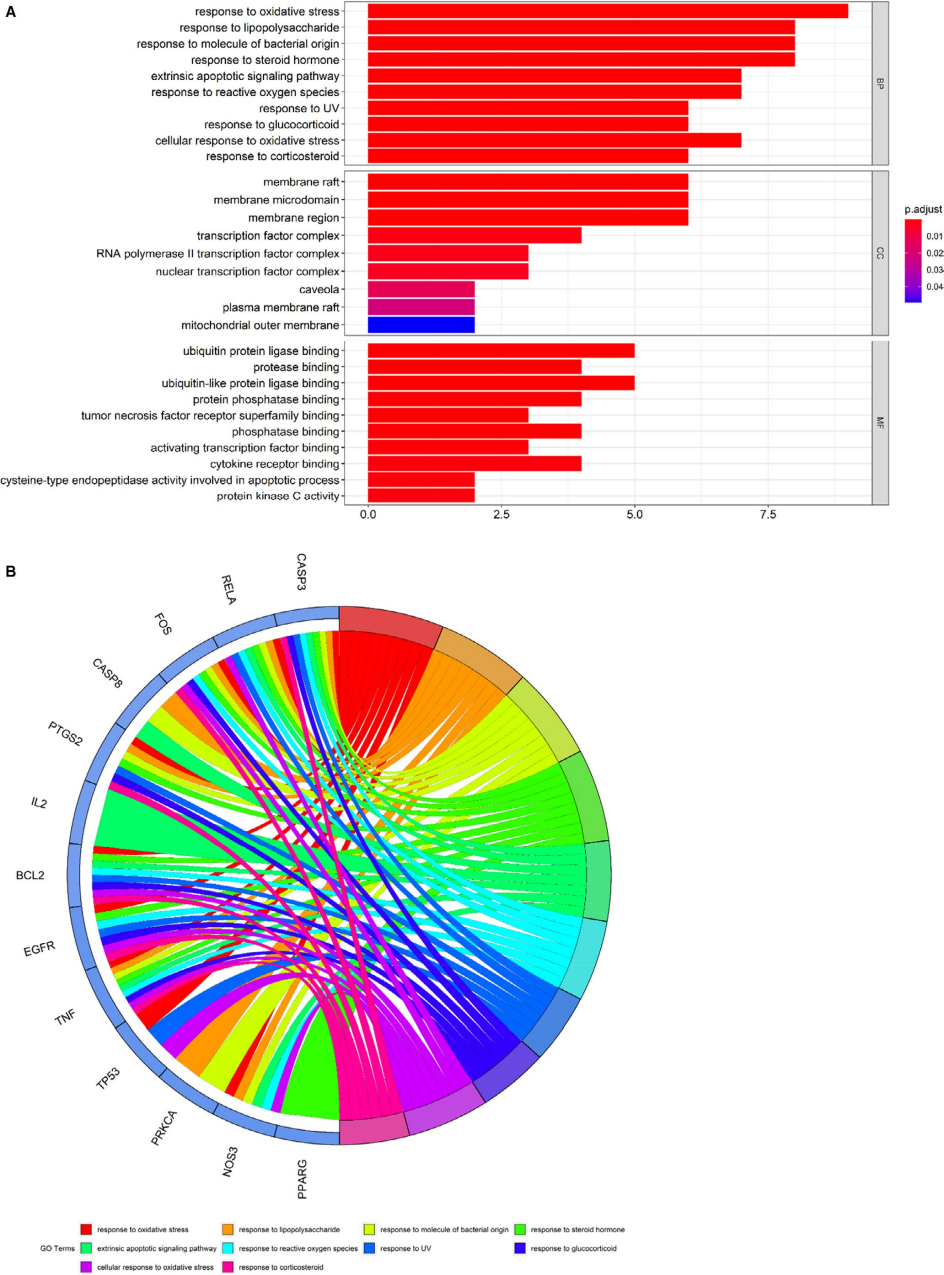
All top biological processes of puerarin to treat COVID‐19 based on enrichment analyses were revealed and visualized. These main functions of puerarin‐anti–COVID‐19 were associated with regulation of different BPs of coronavirus, as detailed in bar diagram (A), circle diagram (B)

**FIGURE 4:**
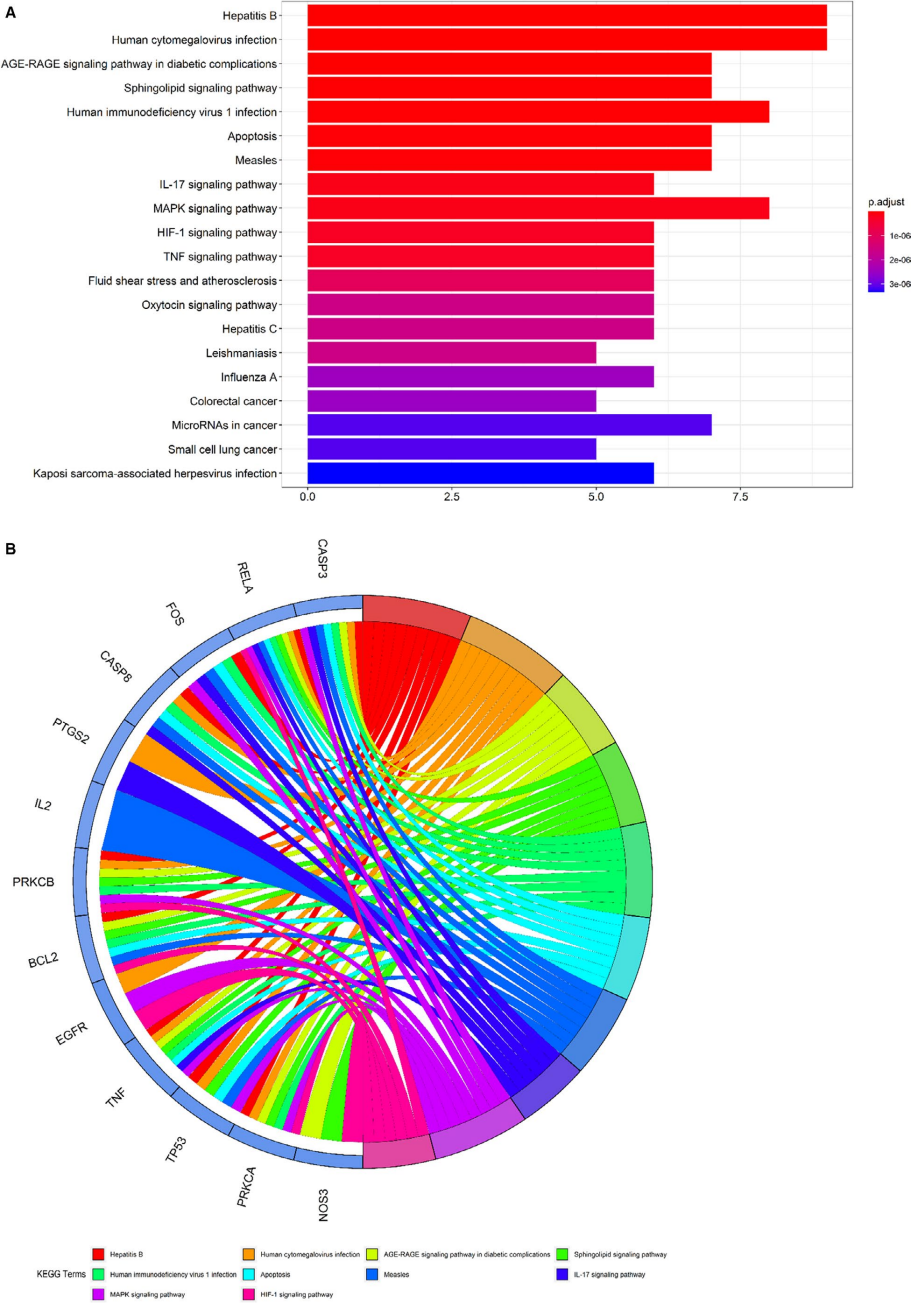
All top molecular pathways of puerarin to treat COVID‐19 from enrichment analyses were uncovered and visualized. These key KEGG pathways of puerarin‐anti–COVID‐19 were related to modulation of respective signalling pathways of coronavirus, as detailed in bar diagram (A) and circle diagram (B)

Following the assays using R language‐related packages, the anti–COVID‐19 molecular pathways from puerarin‐achieved pivotal targets were determined and revealed accordingly. Results showed that puerarin treatment of COVID‐19 targets related biological processes mainly involved in response to oxidative stress, response to lipopolysaccharide, response to molecule of bacterial origin, response to steroid hormone, extrinsic apoptotic signalling pathway, response to reactive oxygen species, response to UV, response to glucocorticoid, cellular response to oxidative stress, response to corticosteroid, response to light stimulus, cellular response to external stimulus, response to osmotic stress, response to mechanical stimulus, positive regulation of neuron death, regulation of apoptotic signalling pathway, myeloid cell differentiation, negative regulation of apoptotic signalling pathway, negative regulation of extrinsic apoptotic signalling pathway and reproductive structure development (Figure 3A, Table S2). Meanwhile, the related cell components of puerarin in the treatment of COVID‐19 target were mainly related to membrane raft, membrane microdomain, membrane region, transcription factor complex, RNA polymerase II transcription factor complex, nuclear transcription factor complex, caveola, plasma membrane raft and mitochondrial outer membrane. In addition, the molecular functions of puerarin in the treatment of COVID‐19 targets mainly involved ubiquitin protein ligase binding, protease binding, ubiquitin‐like protein ligase binding, protein phosphatase binding, tumour necrosis factor receptor superfamily binding, phosphatase binding, activating transcription factor binding, cytokine receptor binding, cysteine‐type endopeptidase activity involved in the apoptotic process, protein kinase C activity, death receptor binding, histone kinase activity, RNA polymerase II transcription factor binding, tumour necrosis factor receptor binding, protein phosphatase 2A binding, pivotal promoter sequence–specific DNA binding, actinin binding, pivotal promoter binding, protein self‐association and scaffold protein binding (Figure 3B). The other 138 KEGG pathways (*P*‐adjust < .05) of pivotal targets were identified and were mainly involved in hepatitis B, human cytomegalovirus infection, AGE‐RAGE signalling pathway in diabetic complications, sphingolipid signalling pathway, human immunodeficiency virus 1 infection, apoptosis, measles, IL‐17 signalling pathway, MAPK signalling pathway, HIF‐1 signalling pathway, TNF signalling pathway, fluid shear stress and atherosclerosis, oxytocin signalling pathway, hepatitis C, leishmaniasis, influenza A, colorectal cancer, microRNAs in cancer, small‐cell lung cancer and Kaposi sarcoma–associated herpesvirus infection (Figure 4A,B, Table S3).

### 3.4 Construction of network diagram

**FIGURE 5:**
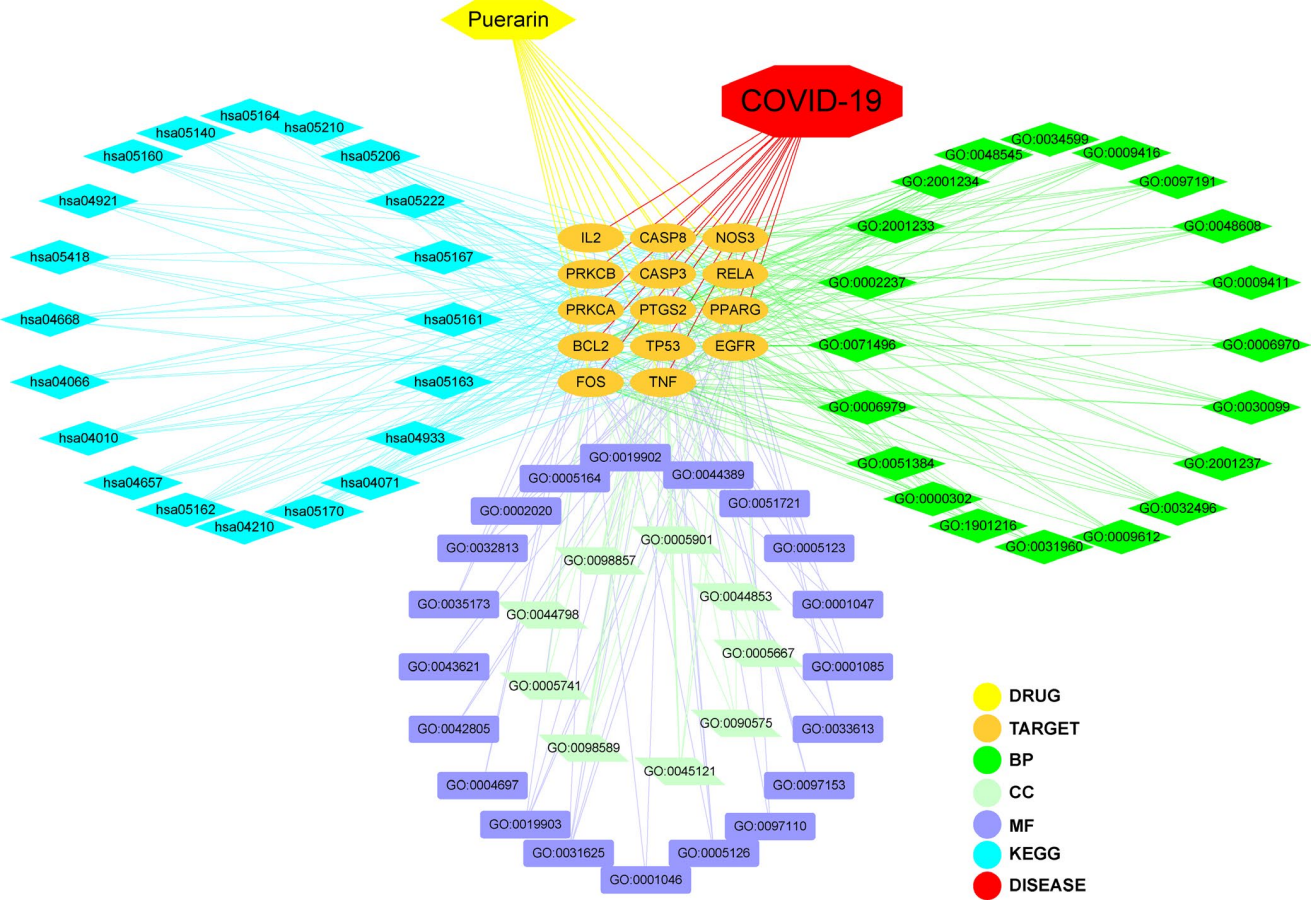
In details, an integrated network map from pivotal targets was plotted and revealed the intersection association of target‐disease‐function‐pathway in puerarin to treat COVID‐19

Further, the network visualization of puerarin‐target‐biological process (BP)‐KEGG‐COVID‐19 was created using Cytoscape 3.7.1 software, as shown in Figure 5.

### 3.5 Molecular docking findings

**FIGURE 6:**
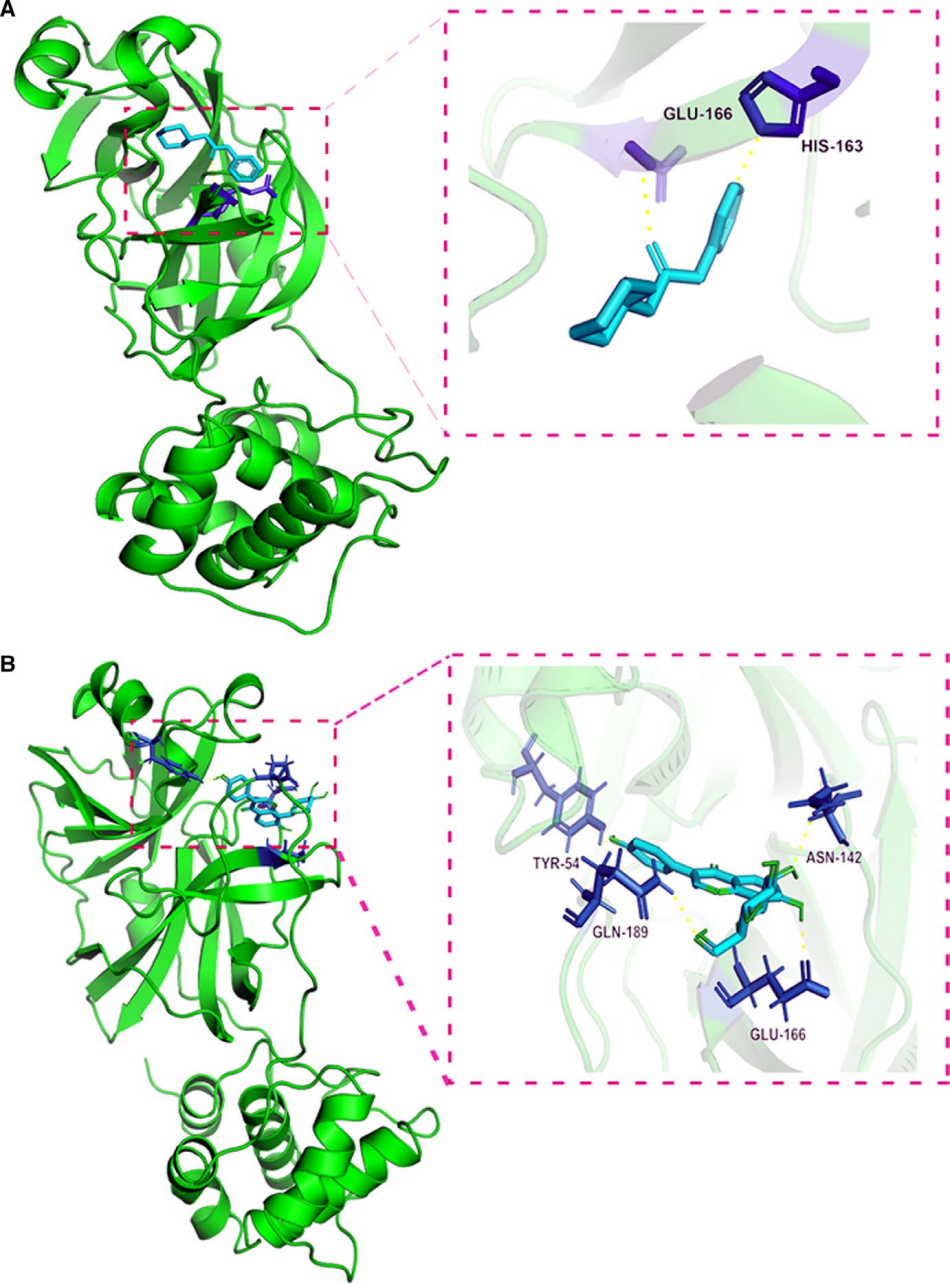
Molecular docking findings suggested that the binding capacity of puerarin against COVID‐19 was significant in the pivotal target/protein. The biostructural original ligands binding to targeting protein with effective docking energy, as shown in A and B

From the interaction diagram of the original ligand and the protein‐ligand complex, the data showed the amino acid residues HIS‐163 and GLU‐166, where the original ligand GWS binds to the 5R84 protein. In the three‐dimensional picture of puerarin, it is observed that the binding residues of hydrogen bonded with TYR‐54, ASN‐142, GLU‐166 and GLN‐189. In addition, puerarin formed hydrogen bonds with the amino acid residues TYR‐54, ASN‐142, GLU‐166 and GLN‐189 in the 5R84 protein (Figure 6). Meanwhile, the free binding energy of puerarin and 5R84 is −7.5 kcal/mol, and the binding free energy of the original ligand GWS and protein is −7.23 kcal/mol. The binding energies of puerarin and GWS were similar. These docking results indicated that puerarin had a potent interaction with 5R84 protein.

## 4 DISCUSSION

In our current study, the in silico findings from the network pharmacology approach and molecular docking analysis revealed the pharmacological targets, functions and signalling pathways of puerarin‐achieved anti–COVID‐19 effects. In detail, all pivotal biotargets of puerarin to treat COVID‐19 were identified via bioinformatic determination, including EGFR, TNF, TP53, CASP3, RELA, FOS, CASP8, PTGS2, IL2, PRKCB, BCL2, PRKCA, NOS3 and PPARG. Molecular docking data indicated that the binding capacity of puerarin with COVID‐19 was significant, indicating the potential and pharmacological activities of puerarin in the treatment of COVID‐19. FOS, a proto‐oncogene, is considered a regulator of cell proliferation, differentiation and transformation. Overexpression of the *FOS* gene is also related to apoptotic cell death.^28^ Prostaglandin‐endoperoxide synthase (PTGS) is responsible for the prostanoid biosynthesis involved in inflammation and mitogenesis when it is induced by specific stimulatory genes.^29^ Some interesting data indicate that targeting suppression of PGE2 synthesis is likely to reduce bacterial infection in mice.^30^ Protein kinase C (PKC), including PRKCB and PRKCA, is involved in diverse cellular signalling pathways, including those involved in cancer.^31^ The proteins encoded by *PRKCB* and *PRKCA* genes have been reported to be associated with numerous cellular functions, such as B cell activation, apoptosis induction and endothelial cell proliferation.^32^ NOS3 (nitric oxide synthase 3) may induce nitric oxide release for the regulation of angiogenesis and cell proliferation.^33^ Interestingly, NOS3 may functionally suppress sepsis‐caused systemic inflammation and myocardial dysfunction in mice.^34^ Collectively, there is no associated investigation for the associations between FOS, PTGS, PRKCB, PRKCA, NOS3 and COVID‐19. Therefore, the current in silico findings using network pharmacology uncovered all pharmacological targets, functions and signalling pathways of puerarin to treat COVID‐19, marked by inhibition of cytonecrosis‐ and inflammation‐associated signalling pathways, such as the IL‐17 signalling pathway, TNF signalling pathway, MAPK signalling pathway and HIF‐1 signalling pathway. Potentially, puerarin may be a promising active compound against COVID‐19. However, it needs to be confirmed by future experimental works.

## 5 CONCLUSION

In silico approaches based on network pharmacology and molecular docking were used to harvest the mapped and pivotal biotargets, pharmacological functions and therapeutic mechanisms of puerarin in treatment of COVID‐19. Additionally, total pivotal biotargets of puerarin to treat COVID‐19 were identified, including new anti–COVID‐19 targets that may comprise FOS, PTGS, PRKCB, PRKCA and NOS3.

## CONFLICT OF INTEREST

None.

## AUTHOR CONTRIBUTIONS

**Xingyue Qin:** Conceptualization (equal); Formal analysis (equal); Investigation (equal); Validation (equal). **Chen Huang:** Data curation (equal); Formal analysis (equal); Methodology (equal); Writing‐review & editing (lead). **Ka Wu:** Resources (equal); Software (equal); Visualization (equal). **Yu Li:** Data curation (equal); Formal analysis (equal); Investigation (equal); Software (equal). **Xiao Liang:** Methodology (equal); Resources (equal); Software (equal). **Min Su:** Funding acquisition (equal); Supervision (equal); Writing‐original draft (equal). **Rong Li:** Conceptualization (equal); Funding acquisition (equal); Project administration (equal).

## Supporting information

Table S1

Table S2

Table S3

## Data Availability

The data that supports the findings of this study are available in the supplementary material of this article.
